# Prophylactic effect of tissue flap in the prevention of bronchopleural fistula after surgery for lung cancer

**DOI:** 10.1007/s00595-024-02927-6

**Published:** 2024-08-28

**Authors:** Tomohiro Habu, Hiromasa Yamamoto, Kentaro Nakata, Kohei Hashimoto, Shin Tanaka, Kazuhiko Shien, Ken Suzawa, Kentaroh Miyoshi, Mikio Okazaki, Seiichiro Sugimoto, Shinichi Toyooka

**Affiliations:** 1 Department of General Thoracic Surgery and Breast and Endocrinological Surgery, Dentistry and Pharmaceutical Sciences, Okayama University Graduate School of Medicine, Okayama, Japan; 2 Department of Thoracic Surgery, Okayama University Hospital, Okayama, Japan; 3 Department of Surgery, Division of Cardiovascular and Thoracic Surgery, Duke University School of Medicine, Durham, NC USA; 4 Organ Transplant Center, Okayama University Hospital, Okayama, Japan; 5 Center for Innovative Clinical Medicine, Okayama University Hospital, Okayama, Japan; 6 Present Address: Center for Clinical Genetics and Genomic Medicine, Shimane University Hospital, 89-1 Enya-cho, Izumo, Shimane 693-8501 Japan

**Keywords:** Bronchopleural fistula, Induction chemoradiotherapy, Covering tissue, Non-small cell lung cancer

## Abstract

**Purpose:**

Bronchopleural fistula (BPF) is a serious complication of lung resection. To avoid BPF, the bronchial stump/anastomotic site is often covered with a flap of surrounding tissue. One risk factor for BPF is radical lung resection after induction chemoradiotherapy for lung cancer. We retrospectively reviewed our database to elucidate the characteristics of tissue flaps that prevent BPF.

**Methods:**

This retrospective study included 152 patients treated between 1999 and 2019. We examined the clinicopathological characteristics, including the type and thickness of the tissue flap used to cover the bronchial stump/anastomotic site, and postoperative complications, including BPF.

**Results:**

BPF occurred in 5 patients (3.3%). All 5 patients had complications that could have affected delayed wound healing, such as pneumonia. The covering tissue flap thickness was significantly greater in patients without BPF than in those who developed BPF (*p* = 0.0290). Additionally, the tissue flap thickness was significantly greater than in those with BPF (*p* = 0.0077), even in high-risk patients who developed pneumonia or radiation pneumonitis on the operative side within 6 months postoperatively.

**Conclusion:**

Perioperative management is crucial to avoid complications affecting the healing of the bronchial stump/anastomotic site, and the covering tissue flap thickness may be an important factor in avoiding or minimizing BPF.

**Supplementary Information:**

The online version contains supplementary material available at 10.1007/s00595-024-02927-6.

## Introduction

Surgical resection remains the standard therapy for early stage non-small cell lung cancer (NSCLC); in Japan during 2018, lobectomy was performed in 31,365 cases (70% of all cases) of primary lung cancer, and bronchopleural fistula (BPF) was the fifth most common cause of surgery-related death (13 of 270) following interstitial pneumonia, pneumonia, cardiovascular events, and respiratory failure after lung cancer surgery [1].

The incidence of BPF is reported to be 1.2–1.6% after lobectomy or bilobectomy for lung cancer without preoperative therapy [2, 3]. However, BPF is reported to be associated with high rates of morbidity (0.8–28%) and mortality (18–50%) [2, 3]. Postoperative BPF occurs most frequently in patients who undergo right pneumonectomy or right lower lobectomy [2, 4, 5], and bronchoplasty is a technical risk factor [3]. Generally, prior steroid therapy, diabetes, chronic obstructive pulmonary disease (COPD), nutritional status, postoperative mechanical ventilation, and postoperative pneumonia are considered to be patient risk factors for BPF [2, 3, 6].

For locally advanced NSCLC (LA-NSCLC), induction chemoradiotherapy (iCRT) followed by surgery is a therapeutic option and is performed by experienced institutions worldwide [7]. In our institution, it has been performed since 1999 [8]. Induction therapy can eradicate systemic micrometastases and prevent microresidual cancer cells, leading to complete resection [9]. In contrast, iCRT increases the risk of subsequent operations because of impairment of the bone marrow function and wound healing [8]. In particular, iCRT reduces bronchial mucosal blood flow; thus, iCRT is significantly associated with the risk of BPF after lung cancer surgery [10]. The frequency of BPF after induction chemotherapy has been reported to increase from 2.1 to 5.8% if radiotherapy is performed concurrently [3, 11]. Radiation pneumonitis is characterized by increased infiltration of neutrophils and macrophages and enhanced activation of proinflammatory and profibrotic cytokines, and is therefore associated with tissue fibrosis, atrophy, and vascular injury [12, 13]. Late effects of radiation can cause fibrotic small vessel disease through radiation vasculopathy [14]; thus, radiation pneumonitis might be a risk factor for BPF.

To prevent the reduction of mucosal blood flow at the bronchial stump/anastomotic site, care should be taken to avoid devascularization of the bronchus during bronchial or lymph node dissection [6]. Additionally, the bronchial stump/anastomotic site is often covered with omentum, pericardial fat pad, thymus, intercostal muscle, pleura, or other tissue [5, 7, 15, 16]. In an animal model, the blood flow of the bronchial stump after pneumonectomy was appropriately reconstituted by the intercostal muscle flap that covered it, relative to a group that did not receive blood supply from the surrounding tissue [17]. Thus, it is of importance to cover the bronchial stump/anastomotic site with well-vascularized tissue, which should help the healing process by providing a source of blood and cells that support the formation of granulation tissue [15, 16]. In a homogeneous cohort of patients who underwent pneumonectomy at the same institution over a 26 year period, it was reported that covering the bronchial stump was a protective factor for BPF [16]. However, the characteristics of the covering tissue that are most effective in preventing BPF remain controversial [6, 18].

The purpose of this study was to evaluate the incidence of postoperative BPF after lung cancer surgery and to identify the risk factors associated with BPF. We particularly focused on patients who underwent iCRT followed by surgery for LA-NSCLC at our institution over the past 21 years. We aimed to determine the optimal tissue for covering the bronchial stump/anastomotic site as a preventive intervention for this complication, considering the relationship between BPF and the thickness of the covering tissue.

## Methods

### Patient selection

This retrospective study was approved by the Ethics Committee, Okayama University Graduate School of Medicine, Dentistry and Pharmaceutical Sciences, and Okayama University Hospital, Okayama, Japan (approval number: Eki1055), and the requirement for written informed consent was waived. Instead, we posted information-disclosing documents with the opportunity to opt out of this study on the website of the Ethics Committee, based on the Ethical Guidelines for Medical and Biological Research Involving Human Subjects, implemented in Japan in June 2021 [19]. Between 1999 and 2019, 182 patients with LA-NSCLC underwent radical resection after iCRT at Okayama University Hospital. The eighth edition of the union for international cancer control (UICC) TNM staging system for lung cancer [20] was used to determine the disease stage and nodal location. The clinical stage was determined as previously described [7, 8].

### Trimodality therapy

While several chemotherapeutic regimens have been applied, cisplatin and docetaxel plus concurrent thoracic radiation with a total dose of 40 or 46 Gy is generally used as the basic regimen for iCRT in our institution, as described previously [21]. The details of the irradiated field for the thorax were previously described. Specifically, the original volume encompassed the primary tumor site with a 2 cm margin around the mass and ipsilateral hilum, as well as the entire mediastinal width [21]. After 2015, prophylactic nodal irradiation was not performed for non-metastatic subcarinal and ipsilateral hilar nodal stations [22]. The surgical procedure was determined according to the extent of the disease, and included lobectomy, bilobectomy, or pneumonectomy, with complete ipsilateral mediastinal and subcarinal nodal dissection.

### Data collection

The clinical data for each patient were collected for the following variables: age at the time of surgery, sex, histology, pretreatment clinical stage, treatment course, operative site, operative procedures, tissue flap used to cover the bronchial stump/anastomotic site, and thickness. All patients were observed until death or March 1, 2020.

### Measurement of the thickness of the covering tissue flap

The thickness of the covering tissue at the bronchial stump/anastomotic site was measured as the shortest vertical distance in the horizontal section of the CT scan. We compared the changes in the thickness of the tissue flap in a subset of the cases in which CT scans were performed within 1 month and 1 year after surgery. Additionally, we compared the tissue thickness of the cases with BPF to that of cases without BPF using CT scans that were performed within 6 months after surgery. Furthermore, regarding high-risk cases for BPF, such as those with pneumonia or radiation pneumonitis on the operative side after surgery, we also compared the cases with BPF to cases in which CT was performed within 6 months after surgery for the thickness of the tissue flap.

### Statistical analyses

All statistical analyses in this study were performed using EZR version 1.54 (Saitama Medical Center, Jichi Medical University, Saitama, Japan), which is a graphical user interface for R version 4.0.3 (The R Foundation for Statistical Computing, Vienna, Austria) [23]. Specifically, the software is a modified version of R commander (version 2.7-1), designed to add statistical functions frequently used in biostatistics. For analyses of continuous values, the t-test or Mann–Whitney U test was used. Statistical significance was set at *p* < 0.05.

## Results

### Patient characteristics

Among 182 patients with LA-NSCLC who underwent radical resection after iCRT between 1999 and 2019, 30 did not undergo a covering procedure. Thus, we reviewed 152 patients. Table 1 summarizes the patients’ clinical characteristics (male, n = 114; female, n = 38; median age, 60 [range 31–79] years). Adenocarcinoma was the most common histological subtype (n = 85; 55.9%). Clinical stage III was the most common (stage IIIA, n = 82, 53.9%; stage IIIB, n = 50, 32.9%). The right side was operated on in 84 patients (55.3%). The bronchial stump/anastomotic site was sutured by hand-stitching in 58 cases (38.2%). The surgical approach in all cases was open thoracotomy, with lobectomy being the most common procedure (110 cases, 72.4%), followed by sleeve lobectomy (17 cases, 11.2%), bilobectomy (14 cases, 9.2%), and pneumonectomy (11 cases, 7.2%).

**Table 1:** Characteristics of patients with locally advanced non-small cell lung cancer undergoing induction chemoradiation followed by surgery with covering of the bronchial stump/anastomotic site (n = 152)

Variables	Results
Age (years)	
Median (range)	60 (31–79)
Sex	
Male	114 (75%)
Female	38 (25%)
Histological subtypes	
Adenocarcinoma	85 (55.9%)
Squamous cell carcinoma	56 (36.8%)
Others	11 (7.2%)
Pretreatment clinical stage (UICC 8th edition)	
IIA	10 (6.6%)
IIIA	82 (53.9%)
IIIB	50 (32.9%)
IIIC	6 (3.9%)
IVA	4 (2.6%)
Operative side	
Right	84 (55.3%)
Left	68 (44.7%)
Suturing technique	
Hand-stitch	58 (38.2%)
Stapler	94 (61.8%)
Operation	
Lobectomy	110 (72.4%)
Sleeve lobectomy	17 (11.2%)
Bilobectomy	14 (9.2%)
Pneumonectomy	11 (7.2%)
*High-risk for BPF (n = 50)	
Postoperative pneumonia	24 (15.8%)
Postoperative radiation pneumonitis	26 (17.1%)

*SD* standard deviation, *UICC* union for international cancer control

^*^High-risk for BPF means patients with pneumonia or radiation pneumonitis on the operative side within 6 months after surgery

### Tissue flaps used to cover the bronchial stump/anastomotic site

Supplemental Table 1 summarizes the tissue flaps used to cover the bronchial stump/anastomotic site. The following tissues were used: pericardial fat pad and/or thymus, n = 102 (67.1%); omentum, n = 25 (16.4%); intercostal muscle, n = 20 (13.2%); both pericardial fat pad and intercostal muscle, n = 2; serratus anterior muscle, n = 2; and latissimus dorsi muscle, n = 1. The pericardial fat pad and/or thymus were collected as pedicled flaps in 101 of the 102 cases. We experienced 1 case of gastric outlet obstruction after covering the omentum, in which gastrojejunostomy was required after surgery. Subsequently, gastrostomies and jejunostomies were performed at the time of surgery in almost all cases, in which the omentum was used to cover the bronchial stump/anastomotic site. Although we experienced another case in which the patient presented with gastric outlet obstruction, it was managed conservatively. The choice of covering tissue is usually at the surgeon’s discretion. Recently, however, there has been a growing trend in our institution to use of pericardial fat pads.

### Frequency of BPF and detailed information of each case

BPF was observed in 5 patients (3.3%) (Supplemental Table 2); however, no significant differences in the frequency of BPF were observed in any of the tissues. Table 2 shows the detailed information of each case with BPF. Five patients underwent right upper sleeve lobectomy with the sacrifice of pulmonary artery A6, right middle and lower lobectomy, right middle and lower sleeve lobectomy with the sacrifice of pulmonary artery A2, right upper and S6 sleeve lobectomy, and right middle and S6 sleeve lobectomy; that is, 4 of 5 cases had bronchial anastomosis. The tissue flaps used for covering were the omentum in 2 cases, and the intercostal muscle, pericardial fat pad, and pericardial fat pad plus thymus in the remaining cases. Each case had factors that may have led to BPF. Two cases had the sacrifice of the pulmonary artery branch to the spared lobe, which we previously reported as a possible risk factor for BPF in sleeve lobectomy after iCRT; that is, residual S6 necrosis associated with the sacrifice of A6 occurred in 1 case, and organizing pneumonia and hemorrhagic infarction had been pathologically demonstrated in the residual upper lobe after completion pneumonectomy in another case with the sacrifice of A2 [24]. In addition, we had 1 case with low nutritional intake because of difficulty in oral intake due to the development of esophageal-bronchial fistula during iCRT and 2 cases of postoperative pneumonia. Furthermore, metastasis in lymph node #7 was found preoperatively in 4 of 5 cases, and the subcarinal area was included in the irradiation field in all 5 cases. After BPF, thoracic fenestration was performed in 3 cases, and intrathoracic omentum transposition was performed in 2 cases in which the omentum was spared for the first surgery. However, 4 of these patients died within 3 years after surgery, including 2 who died from acute respiratory distress syndrome and bronchovascular fistula that developed after BPF (Supplemental Table 3).

**Table 2:** Detailed information of each case with BPF

No	Operative procedure	Bronchoplasty	Covering tissue	Irradiation to subcarinal area	The time of BPF onset (POD)	Perioperative factors for anastomotic failure	Perioperative risk factors for BPF
1	Rt upper sleeve lobectomy	Rt main bronchus-intermediate bronchus	Omentum	Yes	5	Sacrifice of pulmonary artery A6	None
2	Rt middle and lower lobectomy	N/A (hand-stitch)	Intercostal muscle	Yes	11	Difficulty in oral intake	None
3	Rt middle and lower sleeve lobectomy	Rt main bronchus-Rt upper bronchus	Pericardial fat pad	Yes	37	Sacrifice of pulmonary artery A2	None
4	Rt upper and S6 sleeve lobectomy	Rt main bronchus-Rt middle and basal bronchus	Omentum	Yes	162	Pneumonia	Diabetes, nutritional status
5	Rt middle and S6 sleeve lobectomy	Intermediate bronchus-Rt basal bronchus	Pericardial fat pad and thymus	Yes	74	Pneumonia	COPD

Risk factors for BPF: prior steroid therapy, diabetes, chronic obstructive pulmonary disease (COPD), nutritional status, postoperative mechanical ventilation

*POD* postoperative day

### Thickness of the tissue flap used to cover the bronchial stump/anastomotic site

**Fig. 1:**
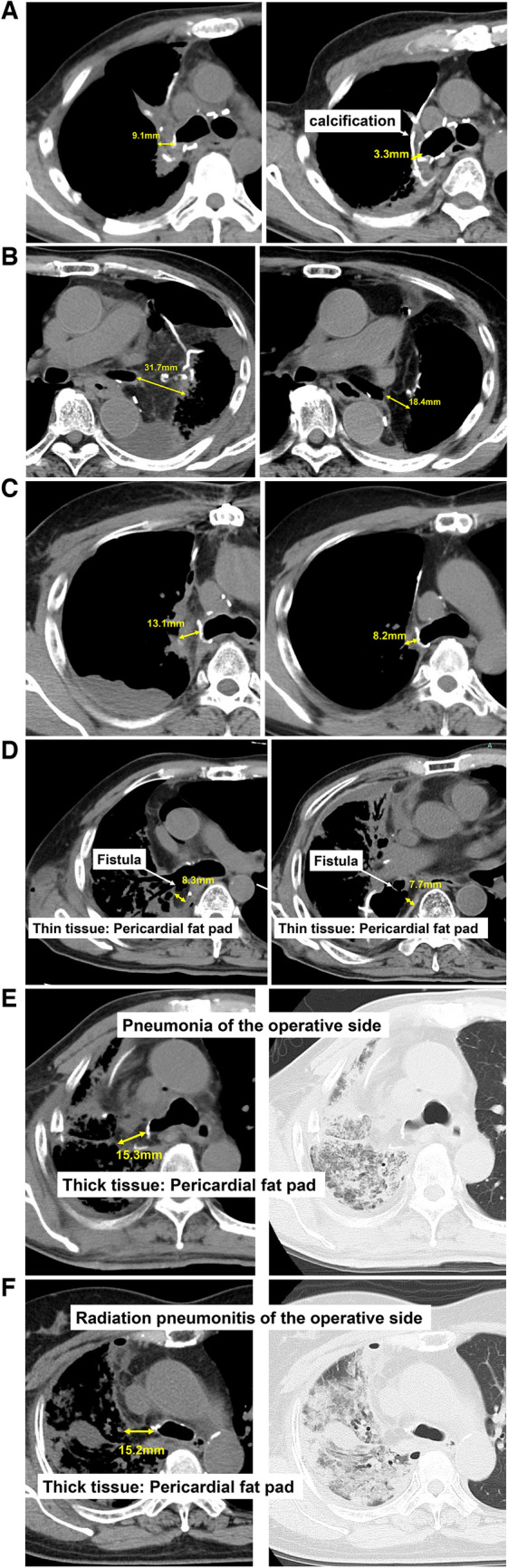
CT scans of patients in whom the bronchial stump/anastomotic site was covered by **A** intercostal muscle, **B** omentum, and **C** pericardial fat pad. The arrow shows the thickness of the tissue flap covering the bronchial stump/anastomotic site in the transverse plane on CT scans obtained within 1 month (left side) and approximately 1 year after surgery (right side). **D** In the cases in which BPF developed (left side: case No.3, right side: case No.5), the thickness of the covering tissue were 8.3 mm and 7.7 mm, respectively. **E**, **F** In the cases without BPF, irrespective of postoperative **E** pneumonia and **F** radiation pneumonitis of the operative side (considered to be risk factors for BPF), the thickness of the covering tissue was 15.3 and 15.2 mm, respectively (left side: mediastinal window setting, right side: lung window setting)

**Fig. 2:**
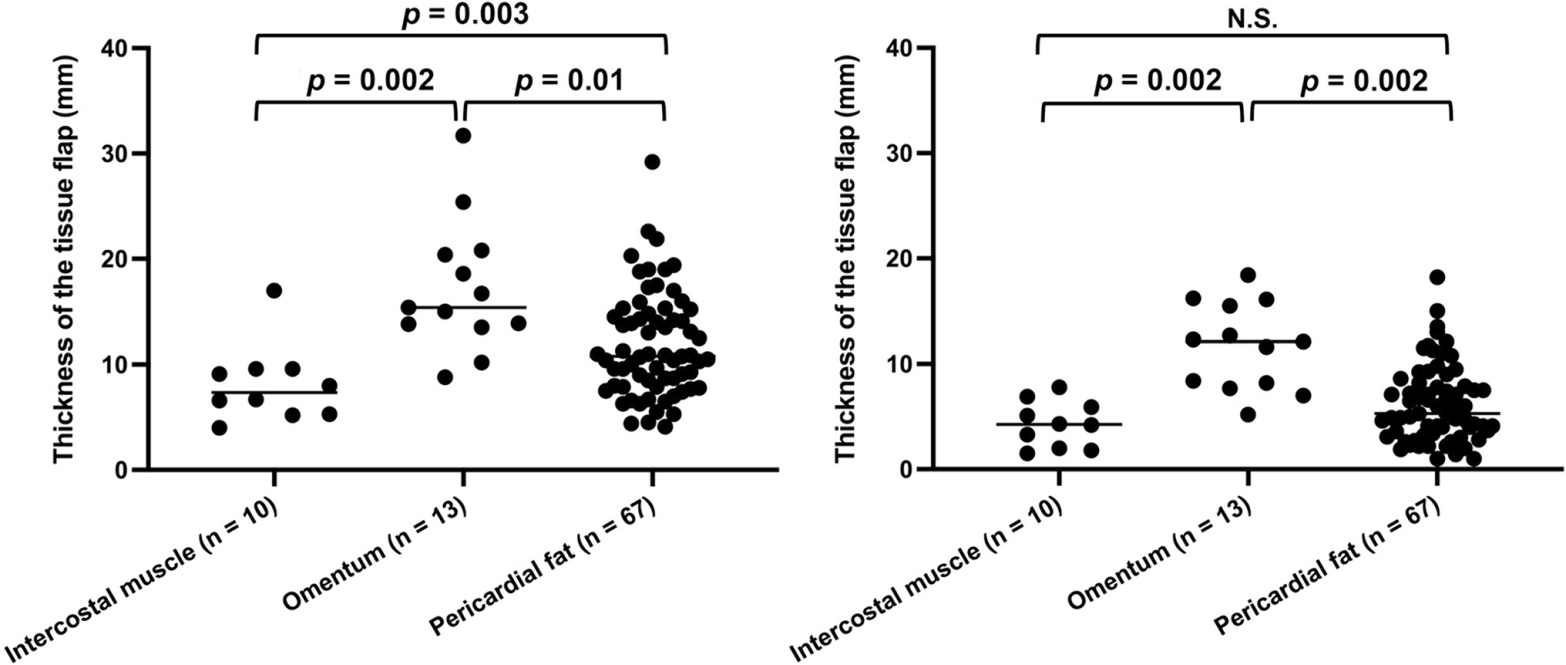
The distribution of the thickness of the tissue flaps covering the bronchial stump/anastomotic site at the time of operation (left side) and 1 year after surgery (right side). We compared the thickness of the intercostal muscle (n = 10), omentum (n = 13), and pericardial fat pad (n = 67)

**Fig. 3:**
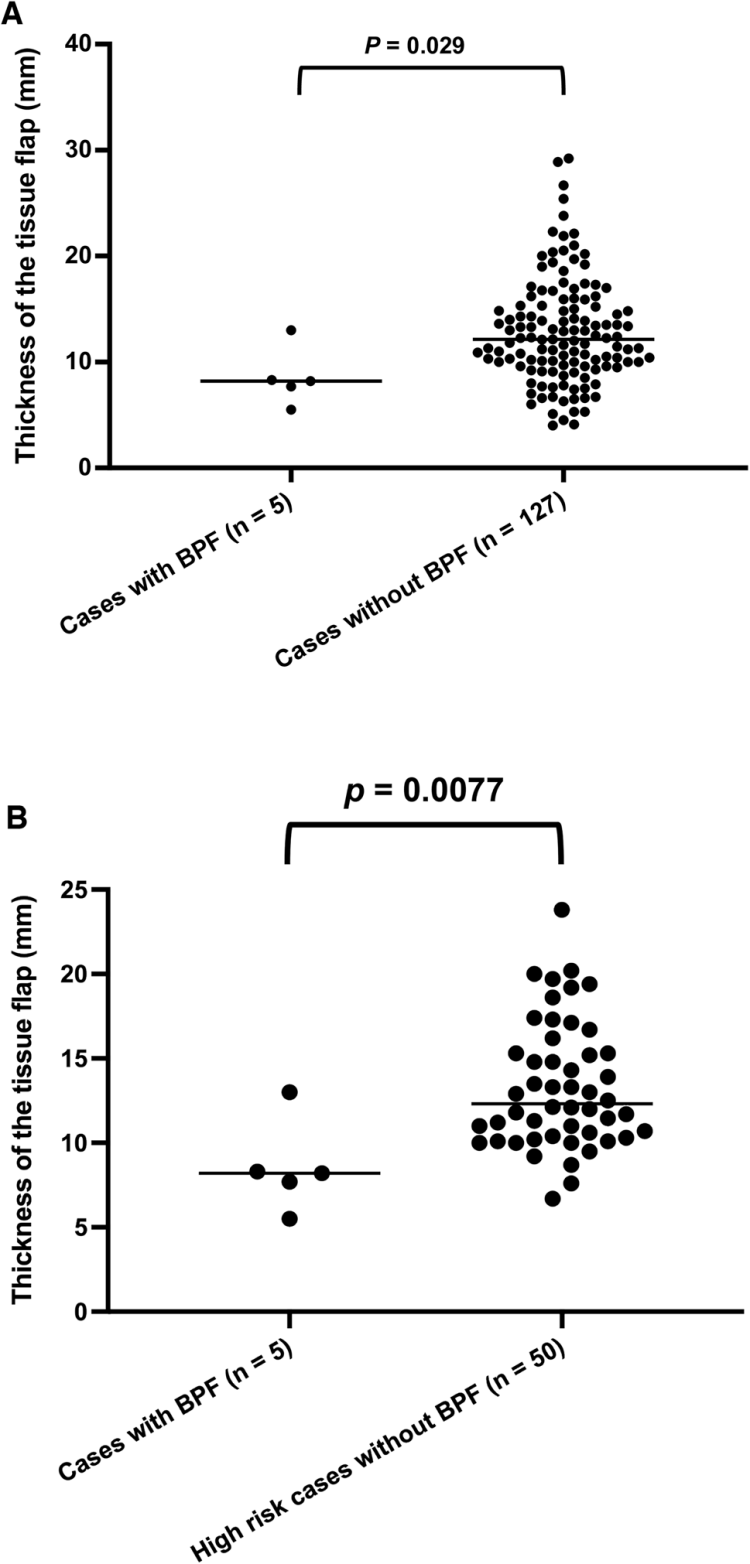
The distribution of the thickness of the covering tissue in **A** cases that developed BPF (n = 5) and the others (n = 127), and in **B** cases that developed BPF (n = 5) and high-risk cases without BPF (n = 50), in which CT scans were obtained within 6 months postoperatively. The thickness of the covering tissue was based on the CT scan at the time at which postoperative complications (e.g., BPF, pneumonia, etc.) developed

We previously encountered a patient with a history of iCRT followed by bilobectomy for LA-NSCLC, who developed BPF after completion pneumonectomy for recurrence [4]. We treated the patient conservatively with N-butyl-2-cyanoacrylate under bronchoscopy because the covering tissue was thick, and the fistula was small (Supplemental Fig. 1). Therefore, we focused on the thickness of the covering tissue in this study. We measured the thickness of the intercostal muscle, omentum, and pericardial fat pad, which are frequently used for covering, in a horizontal CT section in the available cases (n = 90) in which CT was performed within 1 month (Fig. 1A–C left side) and approximately 1 year (Fig. 1A–C right side) after surgery. The measurement was the shortest vertical distance of the covered tissue at the bronchial stump/anastomotic site. Figure 2 shows the distribution of the thickness of the intercostal muscle (n = 10), omentum (n = 13), and pericardial fat pad (n = 67). At the time of the operation, there was a significant difference in tissue thickness among the 3 groups, with the omentum being the thickest. There was no significant difference between the intercostal and pericardial fat pads at one year after surgery. In the cases that developed BPF, the covering tissue appeared to be slightly thin, as shown in (Fig. 1D) (cases 3 and 5). In contrast, there were some patients without BPF, irrespective of the presence of postoperative pneumonia or radiation pneumonitis on the operative side. The covering tissue in these cases appeared to be slightly thicker than that in the cases that developed BPF Fig. (1E, F). Thus, we also analyzed the relationship between the thickness of the covering tissue and the BPF. As shown in (Fig. 3A), when comparing the 127 cases with CT scans obtained within 6 months postoperatively, the thickness of the covering tissue was significantly greater in cases that did not develop BPF (median, 12.1 [range, 4.0–29.2] mm) relative to cases that developed BPF (median, median, 8.2 [range, 5.5–13.0] mm) (*p* = 0.0290). Furthermore, as shown in (Fig. 3B), even among the 50 cases in which BPF did not develop, irrespective of the presence of pneumonia or radiation pneumonitis on the operative side within 6 months after surgery, the thickness of the covering tissue (median, 12.3 [range, 6.7–23.8] mm) was abundant relative to the 5 cases with BPF (median, 8.2 [range, 5.5–13.0] mm) (*p* = 0.0077). As shown in (Table 3), there were no significant differences in perioperative risk factors for BPF between cases with and without BPF. However, in BPF cases, squamous cell carcinoma was histologically predominant, and all cases were managed using hand-stitch procedures.

**Table 3:** Background of patients with locally advanced non-small cell lung cancer undergoing induction chemoradiation followed by surgery with coverage of the bronchial stump/anastomotic site according to the presence or absence of BPF

Variables	With BPF (n = 5)	Without BPF (n = 127)	*p* value
		*High-risk for BPF (n = 50)	
Age (years)	64.8 ± 6.38	60.0 ± 9.25	0.256
		60.2 ± 8.70	0.258
Sex			
Male/female	4 (80%)/1 (20%)	95 (74.8%)/32 (25.2%)	1.000
		35 (70.0%)/15 (30.0%)	1.000
Smoking history (pack-years)			
< 30/ > 30	0/5 (100.0%)	39 (30.7%)/88 (69.3%)	0.321
		19 (38.0%)/31(62.0%)	1.000
Histological subtypes			
Adenocarcinoma/squamous cell carcinoma/others	0/4 (80%)/1 (20%)	77 (60.9%)/43 (33.9%)/7 (5.5%)	0.007
		27 (54.0%)/22 (44.0%)/1 (2.0%)	0.015
Pretreatment clinical stage (UICC 8th edition)			
IIB/IIIA/IIIB/IIIC/IVA	0/4 (80%)/1 (20%)/0/0	8(6.3%)/69 (54.3%)/40(31.5%)/6 (4.7%)/4 (3.1%)	0.835
		5 (10.0%)/26 (52.0%)/16 (32.0%)/1 (2.0%)/2 (4.0%)	0.841
LN metastasis			
N0/N1/N2/N3	0/1 (20%)/4 (80%)/0	14 (11.0%)/22 (17.3%)/80 (63.0%)/11(8.7%)	1.000
		5 (10.0%)/9 (18.0%)/34 (68.0%)/2 (4.0%)	1.000
Suturing technique			
Hand-stitch/stapler	5 (100%)/0	44 (34.6%)/83 (65.4%)	0.006
		22 (44.0%)/28 (56.0%)	0.023
Perioperative risk factors for BPF*			
None/one risk factor/two or more risk factors	3 (60.0%)/1 (20%)/1 (20%)	71 (55.9%)/43 (33.9%)/13 (10.2%)	0.638
		33 (66.0%)/13 (26.0%)/4 (8.0%)	0.578

^*^High-risk for BPF refers to the 50 cases in which the patient did not develop BPF irrespective of pneumonia or radiation pneumonitis on the operative side within 6 months after surgery

^*^Perioperative risk factors for BPF were as follows: prior steroid therapy, diabetes, chronic obstructive pulmonary disease (COPD), nutritional status, postoperative mechanical ventilation

## Discussion

We experienced 5 cases (3.3%) of BPF after iCRT followed by surgery with the covering procedure that is used at our institution. Considering that all of the cases reviewed for the present study (n = 152) underwent chemoradiation, our results are considered to be in line with previous reports.

One of the reasons for covering the bronchial stump/anastomotic site is to maintain blood flow and supply various anti-inflammatory or angiogenic cytokines, which are important for healing of the bronchial stump/anastomotic site [17, 25]. For example, the omentum or pericardial fat pad delivers angiogenic factors that have been proven to improve rapid revascularization of the bronchial stump in animal models or in vitro [25–27]. Another reason is that the volume of the covering tissue can fill the residual space of the thoracic cavity after lung resection. The omentum has a higher volume than the other tissues. Therefore, the omentum potentially reduces dead spaces and adheres to relatively inaccessible high-risk anastomoses [28]. The omentum can also separate the bronchial stump/anastomotic site, mainly from the pulmonary artery, which prevents critical bronchovascular fistula [5]. In contrast, a free pericardial fat pad can remain 6 months after the operation despite the absence of blood supply, which may be due to the angiogenic ability of the pericardial fat pad [26, 29]. In basic experiments, the intensity of neovascularization induced by the pericardial fat pad was reported to be the same as that induced by omentum [26, 29, 30].

At our institution, no significant difference was observed in the incidence of BPF in the covered tissues. However, the thickness of the covering tissue tended to be lower in BPF cases. In patients who did not develop BPF despite postoperative complications, such as pneumonia, thicker tissue was used for covering. Regarding the thickness of each covering tissue, the omentum was significantly thicker than the pericardial fat pad (*p* = 0.002) and intercostal muscle (*p* = 0.01), and the pericardial fat pad tended to be thicker than the intercostal muscle, although the difference was not significant. Even if BPF develops, if the fistula is relatively small and the thickness of the covering tissue is sufficient, there is a chance for it to be cured because covering tissue of sufficient thickness separates it from the pleural cavity, and the blood supply of the covering tissue promotes wound healing [5]. However, in the 5 cases of BPF that we experienced—no matter how rich in blood flow and volume of the covering tissue was—BPF may not have been prevented because the critical complications described in the results may have a negative impact on the healing of the bronchial stump/anastomotic site beyond the healing capability of the covering tissue. In contrast, 1 patient who underwent right upper sleeve lobectomy, in which the bronchial anastomotic site was probably covered with relatively thick tissue, considering the findings of the CT scan that was obtained 6 months after surgery (Supplemental Fig. 2), did not develop BPF despite the presence of pneumonia after the sacrifice of the pulmonary artery that should have been preserved, suggesting that thick tissue may contribute to the prevention of BPF. It is important to minimize the complications that may affect wound healing during the perioperative period, and covering the bronchial stump/anastomotic site with thick tissue may have a significant preventive effect against the development of BPF.

The present study was associated with several limitations. First, this was a retrospective study; therefore, the type of covering tissue was not randomly assigned. Second, the thickness of the covering tissue was examined in the horizontal section of the CT in all cases in the present study; however, this may not always reflect the maximum diameter. Third, the sample size of this study was relatively small, especially for patients with BPF.

In conclusion, complications that affect delayed wound healing at the bronchial stump/anastomotic site should be avoided to prevent BPF in cases of iCRT followed by surgery for LA-NSCLC. However, if such complications occur, the thickness of the covering tissue is probably an important factor in avoiding or minimizing BPF.

## Supplementary Information

Below is the link to the electronic supplementary material.

Supplementary file1 (TIF 2727 KB)

Supplemental Fig 1: Images of the CT scan of the case with a history of iCRT followed by bilobectomy for LA-NSCLC, developing BPF after completion pneumonectomy for the recurrence. We treated this case conservatively by filling the fistula with N-butyl-2-cyanoacrylate (NBCA) under bronchoscopy. Supplementary file2 (DOCX 16 KB)

Supplementary file3 (TIF 3327 KB)

Supplemental Fig 2: An Image of the CT scan of the case in which 6 months have passed after right upper sleeve lobectomy. The bronchial anastomotic site was probably covered with relatively thick tissue considering the findings of CT scan that was taken six months after surgery. Supplementary file4 (DOCX 16 KB)

Supplementary file5 (DOCX 20 KB)

## Data Availability

Data available on request.
