# Supplementary figures and images for: Prophylactic effect of tissue flap in the prevention of bronchopleural fistula after surgery for lung cancer
Source: Surg Today. 2024 Aug 28;55(3):405–13. doi: 10.1007/s00595-024-02927-6 (PMC11842485; doi:10.1007/s00595-024-02927-6)

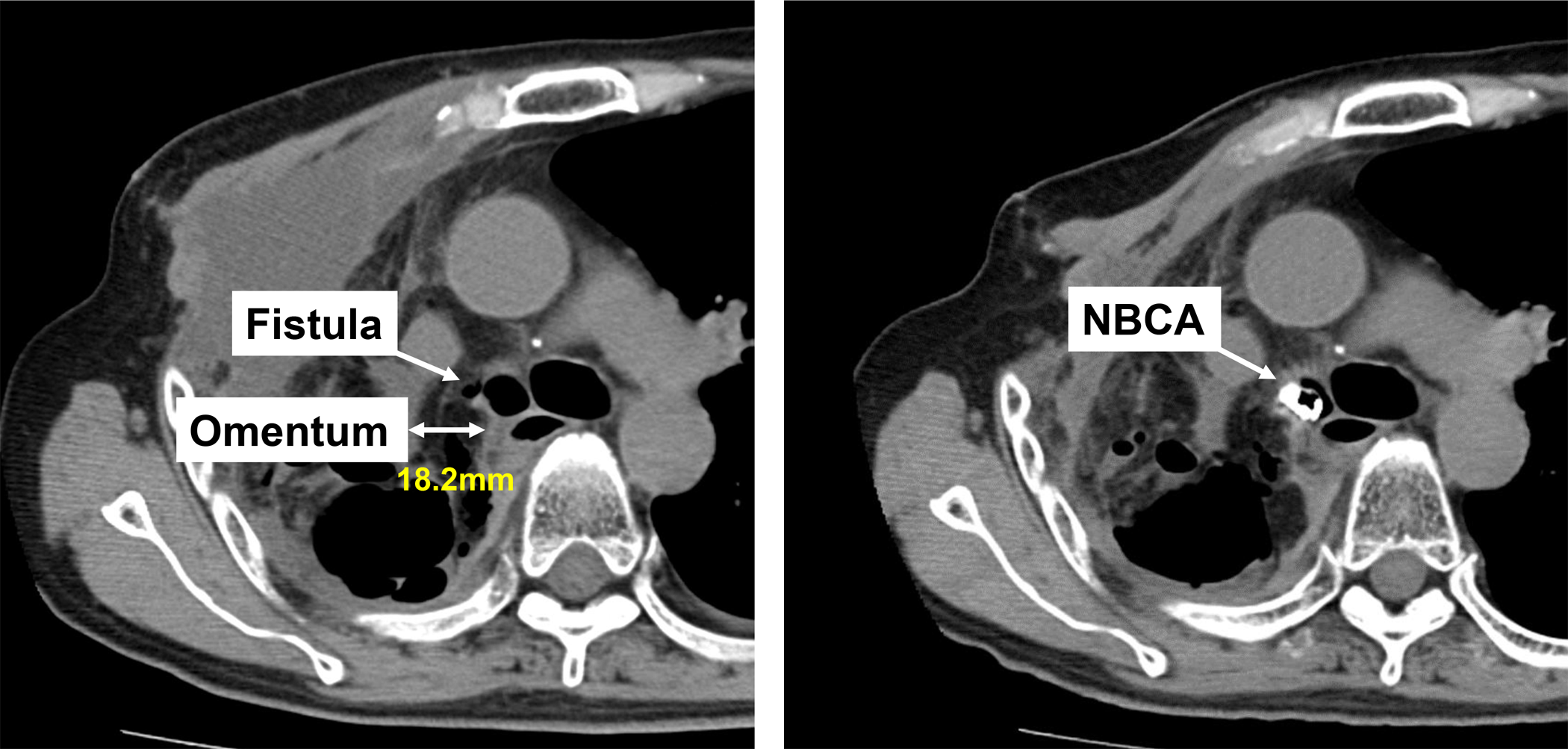

Supplement: Supplementary file 1 — Supplementary file1 (TIF 2727 KB) [file 595_2024_2927_MOESM1_ESM.tif]

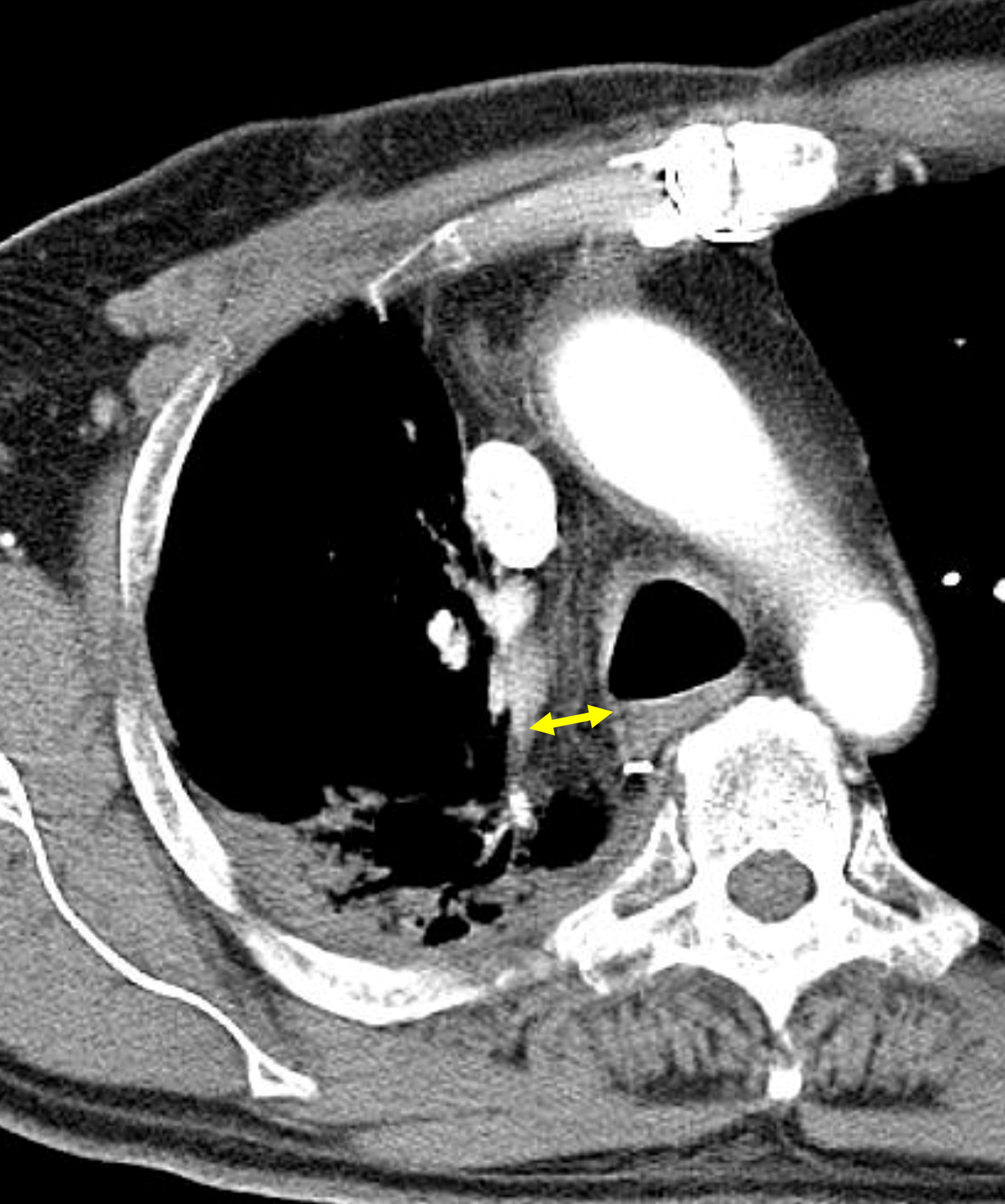

Supplement: Supplementary file 3 — Supplementary file3 (TIF 3327 KB) [file 595_2024_2927_MOESM3_ESM.tif]
