# Supplementary material for: Prophylactic effect of tissue flap in the prevention of bronchopleural fistula after surgery for lung cancer
Source: Surg Today. 2024 Aug 28;55(3):405–13. doi: 10.1007/s00595-024-02927-6 (PMC11842485; doi:10.1007/s00595-024-02927-6)
Supplement: Supplementary file 2 — Supplemental Fig 1: Images of the CT scan of the case with a history of iCRT followed by bilobectomy for LA-NSCLC, developing BPF after completion pneumonectomy for the recurrence. We treated this case conservatively by filling the fistula with N-butyl-2-cyanoacrylate (NBCA) under bronchoscopy. Supplementary file2 (DOCX 16 KB) [file 595_2024_2927_MOESM2_ESM.docx]

Supplemental Fig 1: Images of the CT scan of the case with a history of iCRT followed by bilobectomy for LA-NSCLC, developing BPF after completion pneumonectomy for the recurrence. We treated this case conservatively by filling the fistula with N-butyl-2-cyanoacrylate (NBCA) under bronchoscopy.
