# Supplementary material for: Prophylactic effect of tissue flap in the prevention of bronchopleural fistula after surgery for lung cancer
Source: Surg Today. 2024 Aug 28;55(3):405–13. doi: 10.1007/s00595-024-02927-6 (PMC11842485; doi:10.1007/s00595-024-02927-6)
Supplement: Supplementary file 4 — Supplemental Fig 2: An Image of the CT scan of the case in which 6 months have passed after right upper sleeve lobectomy. The bronchial anastomotic site was probably covered with relatively thick tissue considering the findings of CT scan that was taken six months after surgery. Supplementary file4 (DOCX 16 KB) [file 595_2024_2927_MOESM4_ESM.docx]

Supplemental Fig 2: An Image of the CT scan of the case in which 6 months have passed after right upper sleeve lobectomy. The bronchial anastomotic site was probably covered with relatively thick tissue considering the findings of CT scan that was taken six months after surgery.
