# Supplementary material for: Prophylactic effect of tissue flap in the prevention of bronchopleural fistula after surgery for lung cancer
Source: Surg Today. 2024 Aug 28;55(3):405–13. doi: 10.1007/s00595-024-02927-6 (PMC11842485; doi:10.1007/s00595-024-02927-6)
Supplement: Supplementary file 5 — Supplementary file5 (DOCX 20 KB) [file 595_2024_2927_MOESM5_ESM.docx]

Supplemental Table 1: Tissue used to cover the bronchial stump/anastomotic site

| Pericardial fat pad and/or thymus | 102 (67.1%) |
| --- | --- |
| Omentum | 25 (16.4%) |
| Intercostal muscle | 20 (13.2%) |
| Pericardial fat pad and intercostal muscle | 2 (1.3%) |
| Serratus anterior muscle | 2 (1.3%) |
| Latissimus dorsi muscle | 1 (0.7%) |
| Total | 152 |

Supplemental Table 2: Frequency of bronchopleural fistula (BPF) according to tissue used for covering flap

|  | Total |
| --- | --- |
| Pericardial fat pad and/or thymus | 2/102 (2.0%) |
| Omentum | 2/25 (8.0%) |
| Intercostal muscle | 1/20 (5.0%) |
| Serratus anterior muscle | 0/2 |
| Latissimus dorsi muscle | 0/1 |
| Pericardial fat pad, intercostal muscle | 0/2 |
| BPF | 5/152 (3.3%) |

Supplemental Table 3: Clinical course of each case of BPF

| No | Covering tissue | Treatment for sutural/anastomotic failure | Cause of death | Postoperative  survival (years) |
| --- | --- | --- | --- | --- |
| 1 | Omentum | Completion pneumonectomy > Anastomotic failure  > Thoracic fenestration > Intrathoracic muscle flap transposition | Lung cancer | 4.9 |
| 2 | Intercostal muscle | Intrathoracic omentum transposition  > Thoracic fenestration | Lung cancer | 1.7 |
| 3 | Pericardial fat pad | Completion pneumonectomy  and intrathoracic omentum transposition | Drug-induced acute hepatitis | 0.6 |
| 4 | Omentum | (Died of hemoptysis due to bronchovascular fistula) | Bronchovascular fistula | 0.4 |
| 5 | Pericardial fat pad and Thymus | Residual lower lobectomy, covering the anastomosis  with serratus anterior muscle, and thoracic fenestration | ARDS | 0.7 |

ARDS, acute respiratory distress syndrome
